# Improving the analysis of designed studies by combining statistical modelling with study design information

**DOI:** 10.1186/1471-2105-10-52

**Published:** 2009-02-07

**Authors:** Uwe Thissen, Suzan Wopereis, Sjoerd AA van den Berg, Ivana Bobeldijk, Robert Kleemann, Teake Kooistra, Ko Willems van Dijk, Ben van Ommen, Age K Smilde

**Affiliations:** 1 Dutch nutrigenomics consortium of the Top Institute Food and Nutrition (TIFN), PO Box 557, 6700 AN Wageningen, The Netherlands; 2 TNO Quality of Life, PO Box 360, 3700 AJ Zeist, The Netherlands; 3 Department of Human and Clinical Genetics, Leiden University Medical Center, PO Box 9600, 2300 RC Leiden, The Netherlands; 4 TNO Quality of Life, PO Box 2215, 2301 CE Leiden, The Netherlands; 5 Biosystems Data Analysis, Swammerdam Institute for Life Sciences, Universiteit van Amsterdam, Nieuwe Achtergracht 166, 1018 WV Amsterdam, The Netherlands

## Abstract

**Background:**

In the fields of life sciences, so-called designed studies are used for studying complex biological systems. The data derived from these studies comply with a study design aimed at generating relevant information while diminishing unwanted variation (noise). Knowledge about the study design can be used to decompose the total data into data blocks that are associated with specific effects. Subsequent statistical analysis can be improved by this decomposition if these are applied on selected combinations of effects.

**Results:**

The benefit of this approach was demonstrated with an analysis that combines multivariate PLS (Partial Least Squares) regression with data decomposition from ANOVA (Analysis of Variance): ANOVA-PLS. As a case, a nutritional intervention study is used on Apoliprotein E3-Leiden (APOE3Leiden) transgenic mice to study the relation between liver lipidomics and a plasma inflammation marker, Serum Amyloid A. The ANOVA-PLS performance was compared to PLS regression on the non-decomposed data with respect to the quality of the modelled relation, model reliability, and interpretability.

**Conclusion:**

It was shown that ANOVA-PLS leads to a better statistical model that is more reliable and better interpretable compared to standard PLS analysis. From a following biological interpretation, more relevant metabolites were derived from the model. The concept of combining data composition with a subsequent statistical analysis, as in ANOVA-PLS, is however not limited to PLS regression in metabolomics but can be applied for many statistical methods and many different types of data.

## Background

In the field of life sciences, many studies are performed where the influence of external stimuli is investigated on the organism's gene transcription, protein expression, and metabolism ("systems biology"). Examples of these stimuli are the administration of drugs or a specific diet. These studies aim at understanding how changes in stimuli can affect an individual's genes and health but also at finding biomarkers in tissues or fluids that predict or influence the onset of a disease, or assesses its incidence or patho-physiological behaviour. Measurement of such a marker allows quantification of the extent to which an individual is susceptible to the development of disease.

Successfully analyzing life science studies and so-called omics data (i.e. transcriptomics, proteomics, metabolomics) requires appropriate bioinformatics tools and the conceptual frameworks for analysis and interpretation of large amounts of data generated.

In general, the process from conducting a study to obtaining systems biology data, and deriving molecular insight from that is a delicate one. It requires a well-thought workflow that translates the research hypothesis to a statistical study design, facilitates the performance of validated sample preparation, data acquisition, and the interpretation of statistical results (see also [1]). Another point of attention with this kind of data is that small changes in the stimuli can trigger multiple changes in gene expression, protein and metabolite levels. These changes are usually very small compared to the biological background variation in the data which means that the statistical power of the analysis is low. An additional difficulty in data interpretation is that the data are usually structured. Descending from a biological research question, the data often contain underlying factors such as time, dose, diet, groups, or combinations that correspond to different sources of variation. It can be anticipated that if this data structure is taken into account, the data analysis becomes more focused on relevant sources of variation and therefore has more power. However, most of the currently used statistical approaches simply ignore the structure of the data.

Analysis of Variance (ANOVA) is an obvious method to analyse the data structure by decomposing the total data into different sources of variance. ANOVA is a so-called *univariate *method which means that it analyses one variable at a time. As such, it has been used for the correction or analysis of high dimensional gene expression and metabolomic data [2-4]. In contrast, it was also combined with Principal Component Analysis (PCA) and Simultaneous Component Analysis (SCA) to allow a *multivariate *data analysis while taking the underlying study design into account [5-8]. However, in these approaches, ANOVA was not combined with a supervised analysis such as regression which is a very useful technique in life science studies.

A regression analysis is generally used to determine the relationship between two types of data (e.g. omics data and a phenotype) obtained from the study subjects. In these studies, the aim of regression is not to predict the value of the phenotype but to derive reliable and validated relationships that can be studied further to select and interpret those genes, proteins, or metabolites that are most important with respect to the phenotype. Therefore, this paper aims to show how the quality and interpretability of statistical regression models can be improved by explicitly using the data structure. In the past for the analysis of a chemical process, a like-wise approach was performed by [9], however limited to only within-run and between-run variance.

As a case study, Liquid Chromatography Mass Spectrometry (LC-MS) liver lipidomics data have been used. These data are a part of a large-scale nutritional intervention survey performed in Apolipoprotein E3-Leiden (ApoE3Leiden, [10]) transgenic mice. In these mice, during the early time points, the time resolved development of diet-induced obesity and insulin resistance was investigated. As a phenotype, the hepatic inflammation marker Serum Amyloid A (SAA) was measured in blood plasma of these mice. The final data set consists of 61 mice for which 40 lipids and 19 free fatty acids were measured (i.e. 59 variables) as well as 1 clinical inflammation marker. Inflammation is believed to play an important role in the development of diet related diseases such as obesity, diabetes type II, and atherosclerosis [11] together with the liver as key organ and lipids and free fatty acids as important inflammation related metabolites. For optimal data interpretation, based on the underlying study design, data decomposition by ANOVA is combined with Partial Least Squares (PLS) regression analysis to define the relationship between LC-MS lipidomics and the inflammation marker, SAA. This combination is called ANOVA-PLS. For comparison, standard data analysis was also performed using PLS on the total data. Our findings demonstrate the advantage of incorporating the study design in the data evaluation.

## Methods

### Statistical theory

The principles of ANOVA have been used to separate the different sources of variation of the data based on the underlying study design. The resulting independent data blocks and their combinations can be analysed in various ways including explorative analysis (such as ANOVA-SCA: ASCA) or regression analysis (as in ANOVA-PLS). Before explaining ANOVA-PLS, this section briefly describes the ideas behind ANOVA, SCA, and ASCA.

#### Analysis of Variance

Classical ANOVA techniques can be used to distinguish different sources of variation [12]. The aim of ANOVA is to separate the sources of variation and to assign them to specific factors. This is done by splitting the variations into orthogonal and independent parts. In this paper, an ANOVA model will be used to describe two main factors (*time*, *diet*) and their interaction (*time *× *diet*):

(1) *x*_*kci *_= *μ *+ *α*_*k *_+ *β*_*c *_+ (*αβ*)_*kc *_+ *ε*_*kci*_

where *x*_*kc *_is the data observed for the sample *i *on levels *k *and *c*, *μ *is the overall offset, *α*_*k *_is the model parameter for the factor *time *at level *k*, *β*_*c *_is the parameter for *diet *at level *c*, (*αβ*)_*kc *_is the parameter of *time *× *diet *interaction, and *ε*_*kci *_the residual.

If the parameters of this model are calculated under the proper constraints, the model uniquely separates the total data into orthogonal (independent) data blocks that represent the known factors from the design. The remaining part of the data equals the difference between the sum of the known data blocks and the total data. That part is called the residual part and contains sources of variation that cannot be attributed to a known factor. These sources of variation can originate from unknown factors such as instrumental drift, batch effects, sample work up errors, or measurement errors. In the present data set, the residual part will largely consist of biological variability. This is caused by the fact that each measurement represents a liver sample of a separately sacrificed mouse. Evidently, no true replicates in time are available.

#### Simultaneous Component Analysis (SCA)

SCA [13] is an extension of Principal Component Analysis (PCA; [14]) that can be used in the situation where the same variables have been measured in two or more populations. Examples of such populations are groups of mice that have been fed on different diets. Similar as with PCA, SCA analyses data by summarizing and projecting these in a new space. This new space is defined by so-called simultaneous components, which are linear combinations of the original axis (*i.e*. variables) and that meet some specific mathematical demands. These demands are defined in such a way that the first simultaneous component describes the largest amount of variance possible. Consecutive simultaneous components are orthogonal to all the previous ones and each one describes less variation than the previous simultaneous component. Using the simultaneous components, it is usually possible to describe the data more condensed than originally. This means that a dimension reduction can be performed which facilitates a visual inspection of the data. In a formula, SCA can be represented as follows:

(2) $$X=\left[\begin{array}{c} X_{1}\\ .\\ X_{i}\\ .\\ X_{I} \end{array}\right]=\left[\begin{array}{c} T_{1}\\ .\\ T_{i}\\ .\\ T_{I} \end{array}\right]P^{T}+E$$

where **X **(*N *× *J*) is the matrix of the population matrices **X_i _**(*N*_*i *_× *J*); **T_i _**(*N*_*i *_× *J*) are the scores which are the projected original data into the space of the loadings **P **(*J *× *R*); and **E **(*N *× *J*) is the residual. The indices *N*, *N*_*i*_, *J*, and *R *indicate the total number of objects, the number of objects in population *i*, the number of variables, and the selected dimension in which the data are summarized well (*R *<*J*), respectively. Compared to PCA, the advantage of SCA is that multi-population data are described in one model with one loading matrix containing the simultaneous components. However, if there are no constraints placed on scores **T_i_**, the SCA model of **Equation 1 **is equal to PCA on the concatenated matrix **X**. Timmerman & Kiers [15] describe some possible choices for these constraints and the resulting consequences for the corresponding analysis.

As discussed before, a drawback of both PCA and SCA is that the information sources in the models are confounded. This means that the interpretation of the models can become problematic. A way to solve this problem is by analysing separate data blocks that correspond to unique and known sources of variance.

#### ANOVA-Simultaneous Component Analysis (ASCA)

In ASCA, the advantages of SCA and ANOVA are combined [5,6]. This leads to a method in which the original data are split into orthogonal data blocks that can be attributed to a specific factor of interest. On these separate data blocks, SCA can be applied to analyze them using the concept of data reduction. The multivariate analogue to the univariate ANOVA model (**Equation 1**) is:

(3) *x*_*kcij *_= *μ*_*j *_+ *α*_*kj *_+ *β*_*cj *_+ (*αβ*)_*kcj *_+ *ε*_*kcij*_

In this equation, the index *j *is added to account for all the *J *variables in the data sets that are described. Thus, **Equation 3 **represents a series of *J *ANOVAs. Subsequently, all the terms in the latter equation can be collected in matrices ***X ***with dimensions (*n *× *J*), leading to **Equation 4**:

(4) ***X ***= **1*m*^*T *^+ *X*_*k *_+ *X*_*c *_+ *X*_(*kc*) _+ *X*_*e*_**

where ***1 ***is a size *n *column vector and ***m***^*T *^a size *J *row vector containing all estimates of *μ*_*j*_. If SCA component models are used to approximate the information in the matrices *X*_*k*_, *X*_*c*_, *and X*_*kc*_, the ASCA model that corresponds to the ANOVA model of **Equation 1 **is:

(5) $$X=1m^{T}+T_{k}P_{k}^{T}+T_{c}P_{c}^{T}+T_{\left(kc\right)}P_{\left(kc\right)}^{T}+E$$

The matrices ***T*_*k*_**, ***T*_*c*_**, ***T*_(*kc*) _**are the SCA scores of the factors *K*, *C*, and their interaction, respectively; ***P*_*k*_**, ***P*_*c*_**, and ***P*_(*kc*) _**are the corresponding loadings, while ***E ***is the residual data that cannot be attributed to a known factor. Under the proper constraints ASCA can be calculated very simply. For a balanced design, it has been found that this can be achieved by a proper centring and performing PCA on the rearranged data blocks [5]. Because these constraints are used in this study, in the remainder of this paper the expression 'principal components' will be used instead of 'simultaneous components'.

#### ANOVA-Partial Least Squares

Similar as with ASCA, ANOVA-PLS is the combination of variance decomposition to extract different effects and a subsequent statistical analysis. In this case this analysis is regression with PLS. PLS has been described extensively by [16] and, more recently, for genomic data by [17]. It is a data modelling technique that is used to determine the relationship between a multivariate data set and a univariate phenotype. Depending on the fact if the phenotype is discrete (e.g. a group membership) or continuous (e.g. a concentration), this becomes a classification or a regression analysis, respectively. In this paper, only regression is considered. PLS is able to analyze large numbers of variables in small sample sizes by reducing the dimensionality of the data. The dimension reduction is achieved by constructing latent components (PLS factors), in such a way that these components have maximal covariance with the outcome variable whereas the latent components themselves are uncorrelated. Note that the optimal number of PLS factors is a model meta-parameter that needs to be estimated independently from the regression performance. This is explained in the next section.

For ASCA, each data block was analysed separately by SCA to interpret the different effects. ANOVA-PLS slightly differs in the sense that different *combinations *of effects are used to determine the relation between the data types rather than single effects. The advantage of analyzing selected combinations of effects is that certain effects are highlighted or excluded, compared to the total data, which enables a specific zoom into the data. An additional statistical reason to use effect combinations instead of single effects is that the rank of single effects is too low to build a reliable regression model. This originates from the ANOVA principles where effects are represented by corresponding group means instead of individual values.

#### Statistical regression performance and validation

The quality of the regression models is expressed with a Q^2^_CV _value after double cross-validation [18]. The Q^2 ^parameter is defined as follows [19,20]:

(6) Q2=1−PRESSSSYwithPRESS=∑i=1n(yi−y^i)2andSSY=∑i=1n(yi−y¯)2

In this expression, PRESS is the Prediction Error Sum of Squares (the squared difference between the measured, *y*_*i*_, and the predicted value, ${\hat{y}}_{i}$, for each of the *n *observations), while SSY is the Sums of Squared differences between the measured observations and their mean value $\bar{y}$. Note that if the prediction error (PRESS) becomes larger than the sums of squares (SSY), the Q^2 ^value is smaller than zero indicating a badly predicting model. Because the data set contains 12 groups (4 time points and 3 diets, see the experimental section), a 12-fold cross-validation is performed where in each step one unique group is left out. In this approach, the first cross-validation loop is used to determine the optimal meta-parameter (i.e. the number of PLS factors) and the second one to predict the performance of the model, given the selected meta-parameter. Whereas the number of PLS factors and the prediction performance are determined with a double cross-validation, the final regression coefficients are determined on the total data, because here the aim is only to find the regression coefficients that belong to the optimal PLS model without estimating the prediction performance. The corresponding number or PLS factors is determined in a separate single cross-validation. The need for this approach is shown by [21,22]. From the final model, the significant variables are selected based on jack-knifing according to [23,24]. For this reason, the RSD (Relative Standard Deviation) of the regression coefficients was calculated: the standard deviation divided by the mean. Variables which have an RSD < 0.5 are considered to be significant: their mean is larger than 2 times the standard deviation, indicating a 95% confidence interval. Next, with the significant and the insignificant variables, so-called informative and uninformative models are made, respectively [1]. These models are used to confirm the predictive power of the selected and deselected variables.

### Experimental

#### Design of the case study

**Figure 1:**
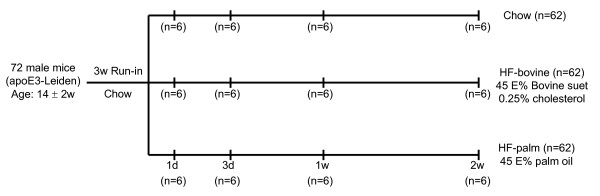
**The design of the study**. This figure shows the division of the mice into the three diet groups and the time points on which the measurements have been performed.

The study design is shown in Figure 1. The study involved 72 male ApoE3-Leiden mice with an age of 14 ± 2 weeks. Three weeks before the start of the study, the mice were fed a standard chow diet. At the start of the study, 24 mice were switched to a High Fat Bovine diet (HF-bovine; 45 energy % bovine lard + 0.25% cholesterol), 24 62 mice to a High Fat Palm Oil diet (HF-palm; 45 energy % palm oil), and 24 mice stayed on the Chow diet. Of each diet group, 6 mice were sacrificed at time points 1 and 3 days, and 1 and 2 weeks. As a consequence of the study design, important factors underlying the data sets are *time*, *diet*, their interaction: *time *× *diet*, and individual (biological) variation.

#### Sample preparation and performed measurements

Liver and orbital blood was obtained from animals after a four hour fasting period (typically from 09.00 to 13.00), it was snap frozen in liquid nitrogen and stored at -80°C until processing. Liver lipidomics data (lipids and free fatty acids, FFA) were analyzed with the Lipid LC-MS [1], and FFA LC-MS TNO platforms which can identify and quantify about 200 different lipids and FFA. For all detected lipids (n = 40) and FFA (n = 19), a relative concentration was calculated (to the internal standard which is lipid or fatty acid class specific). The relative concentrations were corrected for slight differences in liver weight. Serum Amyloid A levels were measured by ELISA specific for SAA (Biosource, see also [25]). Furthermore, quality control (QC) analysis of the analytical measurements was performed on: (1) pooled QC samples, (2) duplicate aliquots of representative samples, and (3) all internal standards in all study and QC samples. Using these data, no instrumental drift or other systematic errors could be detected and the data quality was considered to be good.

#### Pre-processing and imputation

Prior to performing PLS regression analysis, the LC-MS variables of both the fatty acids and the lipids were centred (zero mean) and scaled to unit standard deviation (i.e. auto scaling; [26]). In this way, the individual fatty acids and the lipids will be comparable and have similar scales. Additionally, the SAA measurements were transformed by adding the value of 1 and taking the log_10 _to remove the wide range of the data. This value was added to avoid complications when taking the logarithm of zero or of values very close to zero because this will either be impossible or result in artificially high (negative) values, respectively. It was verified that this pre-treatment did not lead to problems such as the introduction of a problematic bias or the introduction of very large negative values due to the logarithm of numbers very close to zero (data not shown).

Due to missing samples for both the LC-MS metabolites and SAA values, the data are unbalanced. For LC-MS, the data consisted of 65 samples: 5 groups of 5 mice (Chow: 3d; HF-bovine: 3d and 1w; HF-palm: 1d, 1w, and 2w) and 5 groups of 6 mice. For SAA, concentrations were obtained for 63 mice: one group consisted of two mice (Chow, 2w), one group of 4 mice (HF-bovine: 3d), three groups of five mice (Chow: 3d; HF-palm: 1d and 2w) and 7 groups of 6 mice. However, for both two-way ASCA and ANOVA it is beneficial to have on groups that are equally sized (balanced data). This ensures the estimation of independent effects that are crucial for a proper interpretation. Analyzing unbalanced two-way ANOVA can be done in different ways but this is not trivial [27]. Furthermore, special methods like REML (Restricted Maximum Likelihood) might be required. However, the combination of REML with methods for the analysis of high-dimensional data such as SCA is a topic of ongoing research and not available yet. Therefore, in order to deal with the imbalance in ANOVA and ASCA, the groups consisting of less than 6 mice were completed (imputation). For the LC-MS data, imputation was performed by a random draw from a normal distribution defined by the specific group mean and the standard deviation. For SAA, conditional estimations were imputed [28,29]: (1) a PLS model was created between all available pairs of LC-MS metabolites and SAA values (8 PLS factors were used to ensure that both blocks were described for at least 80%) and (2) this model was used to predict the missing SAA values. Consequently, each missing SAA value was replaced by a random draw from a normal distribution defined by the PLS prediction from step 2 as a mean and the overall PLS model residual as a standard deviation. In an experiment where each random draw was randomly repeated 500 times, it will be shown that this approach consistently leads to the same conclusions throughout the paper. According to Rubin & Schenker [30], the strategy adopted is a so-called proper imputation strategy assuming ignorable reasons for the missing data. This means that imputation was performed while reflecting the variability in the data while no systematic differences could be assumed between the missing data within a condition. Note that the aim of this paper is not to present a final prediction model for the study but to show how different analysis approaches (i.e. ANOVA-PLS versus PLS) on the same data can lead to different results.

When applying regression analysis to determine the relationship between liver no imputed samples were used but only those samples that were originally available for both liver lipidomics and blood SAA (61 samples).

#### Software

All statistical data analyses were performed with Matlab 7.1.0, release 14 (The Mathworks, Natick, MA, USA). The used techniques were: PLS in a double cross-validation framework, data decomposition, ANOVA and ASCA. PLS was performed using the PLS Toolbox 3.5.2 (Eigenvector Research, Manson, WA, USA). Double cross-validation was performed according to the software from [21]. Data decomposition, ANOVA, and ASCA were performed according to the software from [6]. The software from these references is available at .

## Results and discussion

### The influence of data imputation

In this study, a conditional imputation strategy was performed to balance the groups, partly relying on a random draw of new samples. This random draw can introduce irrelevant and uninteresting variation which is unwanted. Therefore, in order to evaluate the influence of the random selection, the complete imputation procedure was repeated 500 times. The results were 500 balanced data sets, each consisting of 72 mice with 1 SAA value (phenotype) and 72 lipidomics measurements (i.e. 4 time points × 3 diets × 6 mice per group). For each data set, ASCA, ANOVA and ANOVA-PLS models were made. The results are box plots that show the design-related effect-size differences together with the variation induced by the imputation strategy.

**Figure 2:**
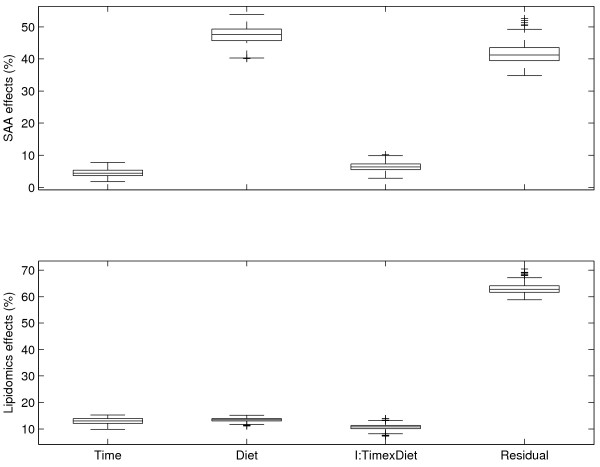
**Box plots of ASCA and ANOVA results**. Box plots are shown for the explained variances of the ASCA (upper plot) and ANOVA effects (lower plot) for 500 random realizations of the imputation strategy.

**Figure 3:**
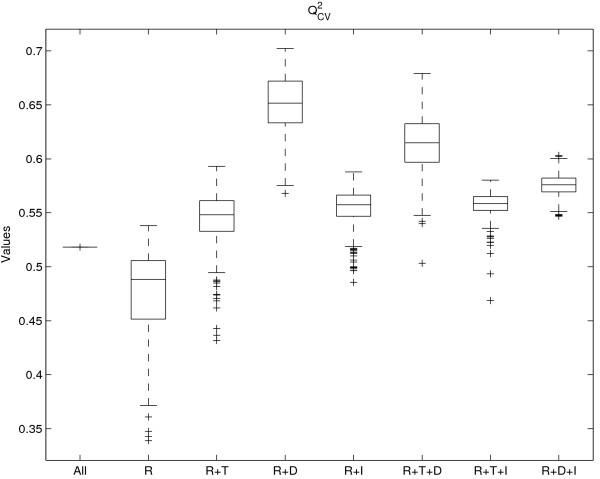
**Box plots of the regression results**. Box plots for the Q^2^_CV _values between the predicted SAA values and the measured ones. Different combinations of the effects are shown at the x-axis. The abbreviations R, T, D, and I stand for: Residual, Time, Diet, and Interaction, respectively.

In Figure 2, it is shown that the overall trend of effect sizes for both ASCA and ANOVA is clear even though the effects of the individual 500 realizations may vary. The Q^2^_CV _values of Figure 3 are derived from the PLS models based on different combinations of the ASCA and ANOVA data blocks. Figure 3 shows a similar situation as Figure 2: the overall trend is clear but individual effect sizes do vary. Together, it can be concluded that the random component of the imputation strategy does not affect the data analysis. Therefore, one of 500 realizations was selected for further analysis in this study. This was the realization which was closest to the median results for the 500 ASCA and ANOVA realizations.

From Figures 2 and 3 it follows that the residual part is a very important component of the total variance. The residual part most likely exists of three elements: (1) lipidomics and SAA measurement noise, (2) ANOVA and ASCA modelling error, and (3) biological variation, possibly as a result of epigenetic effects. However, in this case, it is impossible to further identify and quantify these elements of the residual part because no true replicates could be measured (each mouse was sacrificed). In addition, the underlying factors leading to the biological variation were not known in advance. If these factors would have been known, they could have been treated as the other study design factors, viz. *diet *and *time*. This would make the unexplained residual part smaller and lead to a better understanding of the data structure and to more statistical power. The assumption that structured biological variation is important in the total residual part is supported by the observation that the lipidomic and SAA measurements correlate: a PLS regression model based only on the residual part still performs reasonably well. This is not to be expected if the residual contains only measurement uncertainty in SAA and lipidomics measurements. Therefore, it is likely that the residual is dominated by: (a) structured biological variation, and (b) higher order effects both in SAA and lipidomics.

### ANOVA and ASCA for explorative analysis

As described above, the steps of variance decomposition are used to separate the total data set into blocks that can be attributed to known effects arising from the underlying study design. The advantages of performing regression on the decomposed data are investigated in this paper. However, before doing so, it is very informative to investigate the different effects using ANOVA for the univariate SAA measurements and its multivariate counterpart, ASCA, for LC-MS lipidomics.

**Figure 4:**
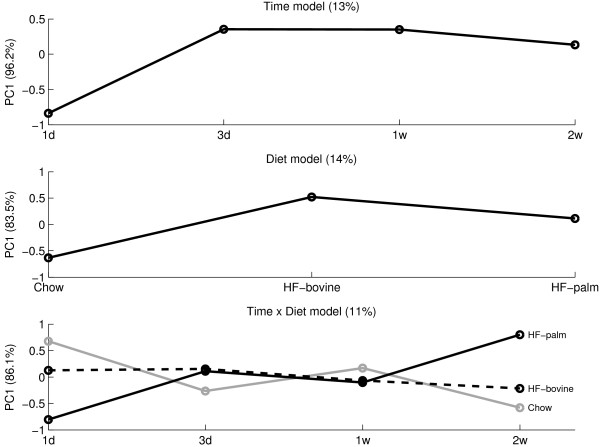
**ASCA results of the lipidomics data**. The ASCA results for the factors *time *(top figure), *diet *(middle figure), and their interaction (bottom figure) in ApoE3Leiden mice liver lipidomics data. In the interaction model, the grey line represents mice on Chow diet, the black line represents mice on HF-bovine diet, and the dotted line represents mice on HF-palm diet. For all effects, the numbers of PCs have been selected to represent at least 80% of the variance of one factor.

Figure 4 shows the SCA results for the lipidomics data set where the factors *time*, *diet*, and *time *× *diet *represent 13% (p = 0.0003), 14% (p = 0.001), and 11% (p = 0.19) of the total variance, respectively. The p-values were derived as described by [31]. It is obvious that from 1d to 3d, the largest time effect is present. Between the other time points, only small differences can be observed. Furthermore, it can be seen that the 3 diets induce different responses although the 2 high fat diets are more similar to each other than to the Chow diet. Finally, because the interaction effect is insignificant, no conclusion can be drawn from that.

**Figure 5:**
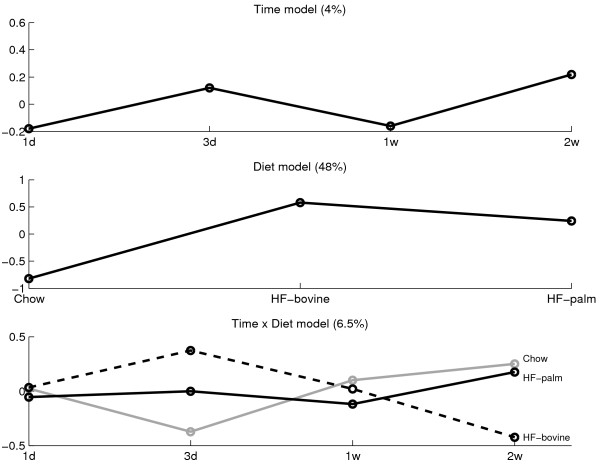
**ANOVA results of the SAA data**. The SAA ANOVA results are shown for the factors time (top), diet (middle), and their interaction (bottom). In the interaction model, the grey line represents mice on Chow diet; the black line represents mice on HF-palm diet; and the dotted line represents mice on HF-bovine diet. The corresponding p-values are 0.13, 9·10^-11^, and 0.17, respectively.

Figure 5 shows the ANOVA results on the univariate SAA measurements. In this model, only the *diet *effect (48%) is significant (p = 9·10^-11^). The diet effect in the SAA measurements shows a similar trend as the diet effect in the lipidomics measurements, even though the latter effect is much smaller. The other effects (*time*: 4%; p = 0.13 and *time *× *diet*: 6.5%; p = 0.17) show a different behaviour for SAA than for the LC-MS metabolites. In addition, these effects have high p-values indicating that they do not reach significance in terms of the traditional cut-offs (α = 0.05 or α = 0.1).

When comparing the ASCA and ANOVA results, the diet effect shows a very similar behavior between the two data sets, and it is the only effect that is significant for both data types. The other effects display different behavior and are not significant for both data types simultaneously.

### Regression analysis with PLS and ANOVA-PLS

The main goal of this study was to investigate the relationship between liver lipid metabolites and plasma inflammation markers by exploiting the underlying study structure in a multivariate analysis. Table 1 shows the regression results on the basis of different combinations of data blocks where each data block contains data for one effect. In Table 2, the correlation coefficients are shown for the regression coefficients from the different models.

**Table 1:** Summary of the regression models

Model nr	Effects included for both the metabolites and for SAA	Q^2^_CV_	PLS factors	Metabolites: Used variation (%)	SAA: Used variation (%)
1	All data	0.52	6	100	100
2	Residual	0.44	4	63	41
3	Residual, Time	0.51	4	75	45
4	Residual, Diet	0.63	5	76	89
5	Residual, Interaction	0.56	4	74	48
6	Residual, Time, Diet	0.63	6	89	93
7	Residual, Time, Interaction	0.56	5	86	52
8	Residual, Diet, Interaction	0.58	6	87	96

The Q^2^_CV _values are presented for the predicted SAA values and the measured ones (n = 61). The prediction of the SAA values was done with double 12-fold cross-validation. The Q^2^_CV _value represents the percentage of variation of SAA that is explained by the variation in metabolites.

**Table 2:** Correlations between the regression models

Model nr	1	2	3	4	5	6	7	8
1	1							
2	0.38	1						
3	0.37	0.97	1					
4	0.79	0.38	0.33	1				
5	0.34	0.95	0.92	0.30	1			
6	0.87	0.34	0.35	0.85	0.25	1		
7	0.37	0.91	0.94	0.26	0.93	0.28	1	
8	0.94	0.40	0.35	0.85	0.38	0.79	0.36	1

The squared correlations are shown for the regression coefficients of the different models. All correlations have a p-value < 0.001.

From these tables it can be concluded that different models are generated when making models on basis of parts of the data. These differences are evident from differences in prediction quality (Q^2^_CV_) and from the correlations between the models. From the correlations, two groups of models appear: (1) the highly correlated models 1, 4, 6 and 8, all containing the diet effect; and (2) the highly correlated models 2, 3, 5 and 7, all without the diet effect. Note that models 1 (all data) and 2 (using only the residual part) are not strongly correlated, indicating that the effects of time, diet, and residual part have an important role. Among these effects, the diet effect is the most important one because it leads to a separation of the models into two groups each containing models that correlate well with each other: the ones with and without this effect. A high correlation can be observed from the correlations between model 2 (only the residual part) and models 3 (residual and time) and 5 (residual and interaction). These models are strongly correlated (R^2 ^≥ 0.95) which implies that adding time and/or interaction to this model does not result in different relations. However, the correlation between these models and model 4 is small (R^2 ^≤ 0.38) which indicates that the modelled relation changes due to the diet effect. This conclusion is supported by the ASCA and ANOVA results: the diet effect is similar in structure and significant for both the lipidomics and SAA measurements. It is important to note that even if the modelled relations strongly correlate, this does not necessarily imply that the predictions by the models are similar. For example, models 1 (all data) and model 4 (residual and diet) highly correlate (R^2 ^= 0.79), but model 4 performs better (Q^2^_CV _= 0.52 versus Q^2^_CV _= 0.63). A similar conclusion can be deduced from comparing models 2 (residual) and 3 (residual and interaction): the correlation is high (R^2 ^= 0.95) but the latter model is superior (Q^2^_CV _= 0.44 versus Q^2^_CV _= 0.56).

Taken together, the best performing models are obtained by removing the *interaction *effect while maintaining the *diet *effect. Including or removing the *time *effect does not affect the prediction performance. Therefore, the relation between liver lipidomics and SAA is determined mostly by the diet. This can also be seen from the ANOVA and ASCA results. Removing the *interaction *might be beneficial for the PLS prediction because this is a higher order effect. In contrast to linear relations, higher order effects cannot be modelled well by PLS. Also, our findings clearly demonstrate that the *residual part *contains an important fraction of the variation that is required to find a good relationship between the lipidomics and the SAA data. It is very likely that this variation contains structured biological variation and/or higher order effects.

For comparison, also univariate correlations have been calculated for all individual metabolites and the SAA measurements, taking into account the design structure in the same way as for the multivariate analysis. The overall most significant correlation (R^2^) was 0.47 (p = 1.13·10^-9^). This indicates that the frequently used one-metabolite-at-a-time approach (i.e. univariate analysis) in this case finds weaker relations between metabolites and SAA compared to a multivariate approach in which the correlations between the separate metabolites are taken into account. Therefore, univariate correlations are not taken into account further.

In the remainder of this paper, only two models will be compared: model 4 (residual and diet) and the original model (model 1). This comparison will demonstrate the differences when interpreting a model that is dedicated towards a specific effect and a model containing all effects. It will be shown that the former model is more reliable and leads to a better interpretation than the original model.

**Figure 6:**
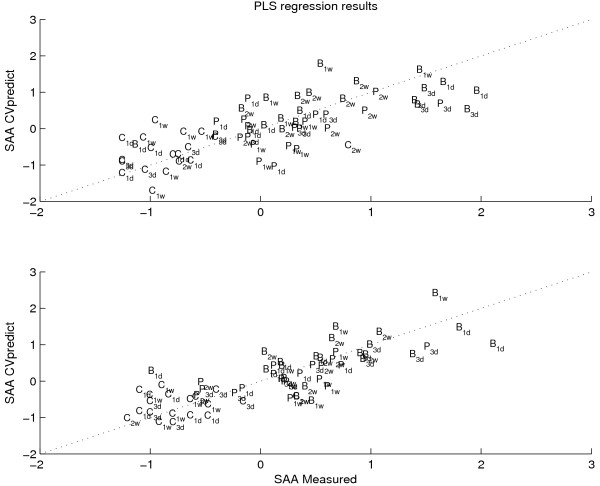
**Regression results of models 1 and 4**. The PLS prediction results are shown for model 1 (Q^2^_CV _= 0.52) and model 4 (Q^2^_CV _= 0.63), respectively. The measured SAA values are plotted against the predicted one. The dotted diagonal line indicates the ideal result with a perfect fit. The diets Chow, HF-bovine, and HF-palm are abbreviated by C, B, and P, respectively.

**Figure 7:**
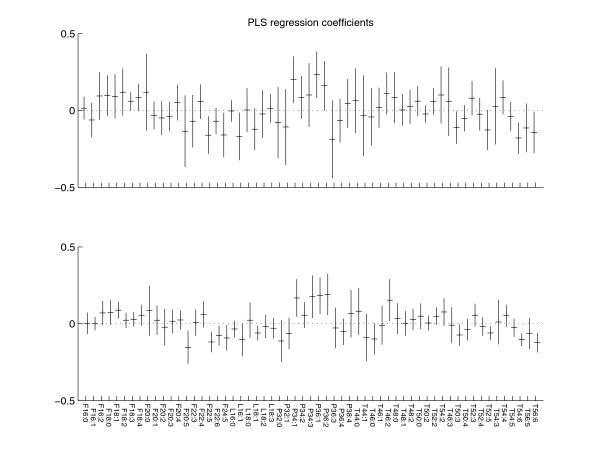
**Regression coefficients of models 1 and 4**. The regression coefficients are shown for models 1 and 4, respectively. The bars show the mean regression coefficients ± 2 × standard deviation.

For the two models, the performances and the final regression coefficients are shown by Figures 6 and 7, respectively. Together with Table 2 it is shown that the similarity is very alike between the two models. However, some differences exist. The confidence intervals of model 4 are smaller than those of model 1 (overall standard deviation of 0.048 versus 0.076). Furthermore, model 1 leads to 8 significant metabolites (metabolites with RSD smaller than 50%) while model 4 leads to 15 significant metabolites. In total, 16 unique metabolites were found significant in either of the two models of which the models have 7 in common. In addition, the following metabolites were never found significant for any of the two models: 14 of 19 FFAs (F16:0, F16:1, F16:2, F18:0, F18:2, F18:3, F18:4, F20:0, F20:1, F20:2, F20:3, F20:4, F22:3, F22:4), 4 of 6 LPCs (L16:0, L18:0, L18:2, L18:3), 6 of 10 PhCs (P32:0, P32:1, P34:2, P36:3, P36:4, P38:4) and 19 of 24 TGs (T44:0, T44:1, T46:0, T46:1, T48:0, T48:1, T48:2, T48:3, T50:5, T50:2, T50:4, T52:2, T52:3, T52:4, T54:2, T54:3, T54:4, T54:5, T56:5). The differences between the significant and insignificant metabolites were not caused by trivialities such as molecular chemical differences as the molecule sizes or the number of saturated bindings. Table 3 shows the significant metabolites for the best model (model 4) and the overlap with model 1.

**Table 3:** Significant metabolites resulting from the regression analysis

Compound	RSD	Compound	RSD	Compound	RSD
F18:1*	0.32	L18:1*	-0.40	T46:2*	0.45
F20:5*	-0.35	P34:1	0.36	T50:3	-0.47
F22:5	-0.28	P34:3*	0.39	T52:5*	-0.36
F22:6*	-0.42	P36:1	0.33	T54:6	-0.20
F24:5	-0.45	P36:2*	0.35	T56:6	-0.26

The significant metabolites and their RSD values on basis of the best regression model (model 4). The compounds marked by an asterisk are not found on basis of the original model (model 1). Additionally, L16:1 was found in the original model but not in model 4 (for which its RSD was 0.52). The absolute RSD values of the insignificant metabolites ranged from 0.51 to 146.00.

Finally, for each of the two models, two new models were made to investigate the robustness of the significant metabolites [1]. For the first new model only the significant metabolites are used (leading to an informative model), while for the other new model only the insignificant metabolites are used (an uninformative model). For the informative and uninformative models based on model 1, the performances decreased to Q^2^_CV _= 0.36 and Q^2^_CV _= 0.34, respectively. For model 4, the informative model performed similar to the original model (Q^2^_CV _= 0.65) while the performance of the uninformative model decreased (Q^2^_CV _= 0.11). This means that the significant metabolites found from model 4 are robust while the insignificant ones are indeed uninformative. For model 1, all metabolites are needed to enable a reasonable model, which means that this model is not suitable to gain interpretation from the models because the reliability of differences between significant and insignificant metabolites is low.

Collectively, our analyses demonstrate that a regression analysis can benefit from data decomposition on basis of the study design which is used to select specific sources of variation (effects) on which a model is built. On the one hand, this can provide more insight into how different effects relate to a phenotype. On the other hand, the regression analysis can improve by better statistics (prediction quality and reliability of the models). It also appears that due to the improved model reliability, more significant variables can be found (more statistical power) which potentially leads to a better understanding of the final model.

### Biological interpretation

After establishing a statistically validated model, the next step is to interpret the model and its important metabolites from a biological perspective. If parts of the model are confirmed by existing knowledge, it becomes more likely that the unconfirmed parts might indicate useful leads that deserve further investigation. However, the interpretation of the most important metabolites from Table 3 is limited by the identifiers that were used. These identifiers are based on the molecular element composition which was again based on the exact mass. Most of these identifiers require additional analysis to uniquely determine the corresponding metabolite name. However, some of the identifiers did allow a unique association with a metabolite name and consecutively lead to a plausible biological interpretation.

F20:5 and F22:6 are known to be unique identifiers for omega-3 fatty acid EPA and omega-3 fatty acid DHA, respectively. Moreover, it is known that F22:5 and F24:5 are also omega-3 fatty acids. Table 3 and Figure 7 show that these fatty acids are negatively correlated with the used phenotype: the inflammation marker SAA. This again corresponds well with the fact that omega-3 fatty acids are known to be anti-inflammatory [32]. Note that, omega-3 fatty acids EPA and DHA (F20:5 and F22:6) are only found in model 4: the model that finds the best and most reliable relation with the phenotype.

## Conclusion

Regression analysis is a statistical tool that can uncover relationships between two types of data sets. Once reliable regression models are derived, regression coefficients can be used to derive knowledge regarding the two types of data. However, when studying complex biological systems, the data comply with a study design. The goal of a study design is to generate relevant information while diminishing the unwanted variation. Knowledge about the study design can be used to decompose the total data into data blocks that are associated with specific effects. Subsequent analysis can benefit from this decomposition if these are applied on selected combinations of effects. In this way more focus can be put on specific blocks and disturbances can be minimized.

This paper shows that combining ANOVA with PLS regression leads to models that differ in structure and statistical quality. The regression coefficients of these different models can then be used to study specific effect related relations between two types of data. Additionally, removing specific effects from the relation can lead to statistical models that are better, more robust and with more reliable important variables. The biological interpretation shows that reliability of the most important variables is important to avoid missing useful information. This is especially the case for nutritional studies where subtle effects are expected.

A potential drawback of this approach is that unbalanced multi-way ANOVA (and ASCA) models are difficult to interpret. In this paper, this problem was solved by a conditional imputation strategy. It was shown that this strategy leads to consistent overall conclusions and therefore did not affect the analysis.

It was also shown that the often used univariate approach (finding correlations between single variables and the phenotype) did not lead to models that were competitive to multivariate regression. The ultimate best univariate model performed much worse than the multivariate one, even though the analyses were performed on the same selection of data sets.

Importantly, the presented approach of PLS regression on selected data blocks is not limited to only metabolomics data. It is applicable to all types of data with a known underlying structure. Moreover, it can be used in studies where one of the data sets is continuous (such as a concentration), for data that contain a subdivision in groups (classification problems) and for explorative analysis of the data (e.g. ASCA).

## Abbreviations

ANOVA: Analysis of Variance; ApoE3: Apolipoprotein E3; ASCA: ANOVA-Simultaneous Component Analysis; CV: Cross-validation; FFA: Free Fatty Acids; IS: Internal standard; LC-MS: Liquid Chromatography Mass Spectrometry; LPC: Lysophosphatidylcholines; PC: Principal Component; PCA: Principal Component Analysis; PhC: Phosphatidylcholines; PLS: Partial Least Squares; RSD: Relative Standard Deviation; SAA: Serum Amyloid A; SCA: Simultaneous Component Analysis; TG: Triglycerides.

Scalars

x: Data sample; μ: Overall offset; α, β: ANOVA parameters; C: Factor of diet; c: Subscript on scalar, vector, or matrix indicating the factor of diet; ε: Residual; J: Number of variables; j: Subscript on scalar indicating a variable j specific value; K: Factor of time; k: Subscript on scalar, vector, or matrix indicating the factor of time; n_i_: Total number of objects (in population i); PRESS: Prediction Error Sum of Squares; R: Reduced dimension in SCA (R < J); SSY: Sums of Squared differences between the true and their mean value; y_i_: Measured value for SAA for sample i; ${\overset{⌢}{\text{y}}}_{\text{i}}$: Predicted value for SAA for sample i; $\bar{\text{y}}$: Mean value of all y_i_;

Vectors and matrices

**1**: Column vector of size n with the value of one; **E**: Residual; **m**: Row vector of size J containing estimates of μ_j_; **P**: Loadings; **T_i_**: Score of population i; **X_i_**: Data matrix of population i; **X**: Data matrix of population matrices **X_i_**

## Authors' contributions

UT defined the topic of the manuscript, performed statistical analysis and drafted the manuscript. SW assisted with the metabolomics analysis and wrote specific sections. SAAvdB performed the mice study and wrote specific sections. IB was responsible for the LC-MS data measurements and wrote specific sections. RK, TK, and KWvD were responsible for the mice study and the SAA measurements. TK and BvO supplied conceptual and biological feedback on the manuscript. AKS supplied conceptual and statistical feedback on the manuscript. All co-authors reviewed draft versions.
